# Resveratrol relieves gestational diabetes mellitus in mice through activating AMPK

**DOI:** 10.1186/s12958-015-0114-0

**Published:** 2015-11-05

**Authors:** Liangqi Yao, Jipeng Wan, Hongyan Li, Jian Ding, Yanyun Wang, Xietong Wang, Mingjiang Li

**Affiliations:** Department of Obstetrics, Shandong Provincial Hospital Affiliated to Shandong University, No. 324 Jingwu Road, Jinan, 250021 Shandong China; Department of Gynaecology, Shandong Provincial Hospital Affiliated to Shandong University, No. 324 Jingwu Road, Jinan, 250021 Shandong China; Maternal and Child Health Hospital of Shandong Province, Jinan, Shandong China

**Keywords:** Resveratrol, Gestational diabetes mellitus, *db/+* mouse model, AMP-activated protein kinase, Glucose-6-phosphatase

## Abstract

**Background:**

Gestational diabetes mellitus (GDM) is a disease often manifests in mid to late pregnancy with symptoms including hyperglycemia, insulin resistance and fetal mal-development. The *C57BL/KsJ-Lep*^*db/+*^ (*db/+*) mouse is a genetic GDM model that closely mimicked human GDM symptoms. Resveratrol (RV) is a naturally existing compound that has been reported to exhibit beneficial effects in treating type-2 diabetes.

**Methods:**

In this study, we investigated the effect of RV on the pregnant *db/+* GDM mouse model, and the underlying molecular mechanism.

**Results:**

RV greatly improved glucose metabolism, insulin tolerance and reproductive outcome of the pregnant *db/+* females. Moreover, we found that RV relieved GDM symptoms through enhancing AMPK activation, which in turn reduced production and activity of glucose-6-phosphatase in both pregnant *db/+* females and their offspring.

**Conclusions:**

Our findings further supported the potential therapeutic effect of RV on not only diabetes, but also alleviating GDM.

**Electronic supplementary material:**

The online version of this article (doi:10.1186/s12958-015-0114-0) contains supplementary material, which is available to authorized users.

## Background

Gestational diabetes mellitus (GDM) is a condition typically caused by insufficient insulin production or signaling in pregnant women [1]. Patients suffering from GDM usually do not present any signs of diabetes prior to pregnancy, and are commonly diagnosed during the second trimester of pregnancy, with 3–5 % of women even suffering long-term diabetes after pregnancy [2]. Apart from maternal diabetic symptoms, GDM also resulted in abnormal fetal development [3]. The *C57BL/KsJ-Lep*^*db/+*^ (*db/+*) mouse, harboring a heterozygous mutation in the leptin receptor gene *Lepr* [4], closely mimicked GDM symptoms observed in human patients. At the non-pregnant state, they exhibited largely normal glucose and insulin tolerance until gravidity [5, 6]. During pregnancy, the *db/+* females presented typical GDM symptoms including hyperglycemia, insulin resistance and obesity. Fetal development was also adversely affected, with increased body weight of offspring at birth [7, 8].

**Fig. 1:**
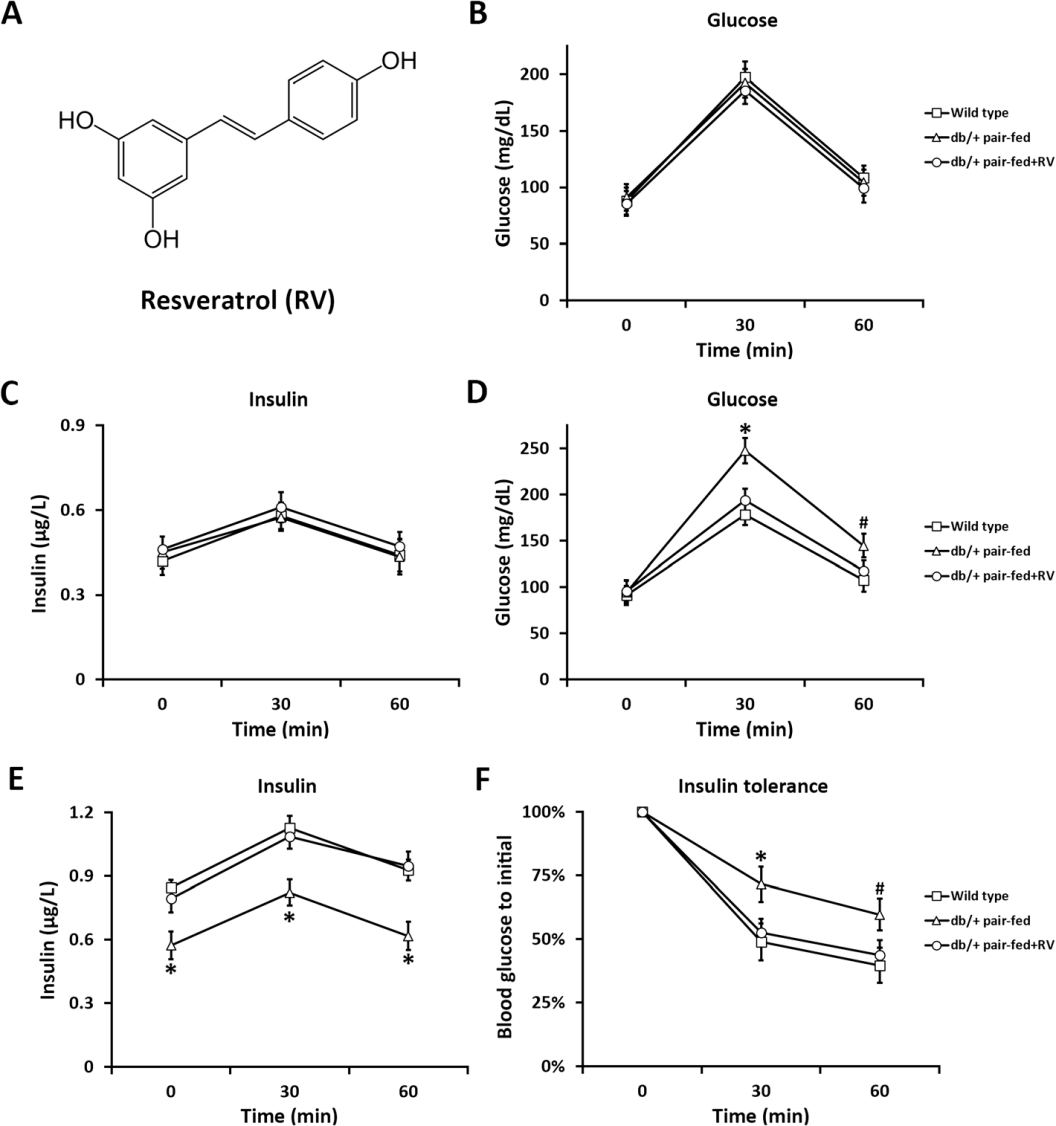
Effects of RV on glucose and insulin tolerance. **a** Chemical structure of resveratrol (RV). **b** and **c** Effects of RV on serum glucose (**b**) and insulin (**c**) levels during intraperitoneal glucose tolerance test in non-pregnant wild type and *db/+* mice. **d** and **e** Effects of RV on serum glucose (**d**) and insulin (**e**) levels during intraperitoneal glucose tolerance test in pregnant wild type and *db/+* mice on gestation day 10. **f** Effects of RV on serum glucose level during intraperitoneal insulin tolerance test in pregnant wild type and *db/+* mice on gestation day 10. Values represented mean ± SEM (*n* = 18). *P < 0.01, ^#^ *P* < 0.05, *db/+* pair-fed vs wild type and *db/+* pair-fed + RV mice at the same time point

Resveratrol (Fig. 1a. 3,4,5-trihydroxy-trans-stilbene, RV) is a naturally existing polyphenol that can be found in grapes, cranberries and red wine, and increasing body of studies have recently reported its role in the treatment of diabetes in various animal models. In diabetic mouse models, either from genetically deficient in insulin receptor substrate 2 or streptozotocin-induced diabetes, RV treatment restores peripheral insulin sensitivity in a sirt1-independent manner [9]. However in streptozotocin-induced diabetic rats, RV was found to regulate the sirt1 pathway and glucose transporters [10]. Using a similar streptozotocin-induced diabetic rat model, Sadi et al. conducted a series of studies, demonstrating that RV induced differential gene expression in the liver that antagonized diabetic symptoms [11], while regulated oxidative biomarkers and anti-oxidant enzymes in the brain [12], and more importantly improved hepatic insulin signaling and reduced inflammatory response [13]. Also in streptozotocin-induced diabetic rats, RV exerted beneficial effects on the kidney through extenuating the oxidative stress and down-regulation of receptor for advanced glycation end-products [14].

Of particular relevance to our current study, RV was found to attenuate high glucose-induced oxidative stress and cardiomyocyte apoptosis through AMP-activated protein kinase (AMPK) [15]. AMPK could be a potential cross-species target of RV function in diabetes, because a recent study employing the Japanese macaque high fat dietary model, it was also found to stimulate AMPK activation and transporter expression in the placenta [16]. The AMPK signaling pathway coordinates cell growth, autophagy and metabolism [17]. Particularly in the liver, AMPK activation reduces glucose production, by phosphorylating and suppressing the class IIa family of histone deacetylases (HDACs), resulting in down-regulation of gluconeogenesis genes such as glucose-6-phosphatase (G6Pase) [18].

In this study, we applied RV on the *db/+* genetic GDM mouse model, aiming to investigate if RV could exhibit similar functions on GDM as reported by previous diabetes-related studies [15, 16]. By measuring serum glucose and insulin levels before and during pregnancy, we could evaluate the effect of RV on GDM-related symptoms. Furthermore we also examined states of AMPK activation, in order to decipher the potential underlying mechanism explaining the beneficial role of RV on GDM.

## Methods

### Animals and study design

Care and use of mice in this study abide the guidelines and protocol approved by the Institutional Animal Care and Use Committee of Shandong Provincial Hospital Affiliated to Shandong University. All efforts were exhausted to minimize unnecessary suffering of experimental mice.

Six to eight weeks old C57BL/KsJ^+/+^ (wild type) and C57BL/KsJ^*db/+*^ (*db/+*) mice were purchased from Jackson Laboratories. Mice were housed in a room with controlled temperature (22 ± 2 °C), humidity (40–60 %) and light cycle (12/12 h light/dark). Mice were fed with the chow diet (Harlan Teklad) consisted of 29 % protein, 47 % carbohydrate and 17 % fat, and water. Female mice were randomly divided into three experimental groups (*n* = 18 per group): wild type, *ad libitum*-fed; *db/+* pair-fed, food intake of the *ad libitum*-fed wild-type was measured daily, and the same amount of food was pair-fed to *db/+* mice; *db/+* pair-fed + RV, *db/+* pair-fed mice were administered by oral gavage with resveratrol (RV; Sigma-Aldrich), solubilized in water, at the dose of 10 mg/kg body weight per day, throughout the entire span of the study. Wild type and *db/+* pair-fed groups of mice were also orally gavaged with water only daily, throughout the entire span of the study, to serve as vehicle control. At 10–12 weeks of age, female mice were individually mated with males of the same genotype, and mating was confirmed by the presence of a copulatory plug the next morning, which was designated gestation day (GD) 0. Since not all females could become pregnant after mating, an initial number of more than 18 females per group were used, and eventually 18 pregnant females were used in each experimental group as described above.

### Glucose tolerance test

Mice before pregnancy and on GD 10 were fasted for 6 h and injected intraperitoneally with glucose at 2 g/kg body weight. Blood samples were collected from the tail using capillary tubes at 0, 30 and 60 min after glucose administration.

### Insulin tolerance test

Mice on GD 10 were fasted 6 h and injected intraperitoneally with insulin at 0.75 U/kg body weight. Blood samples were collected from the tail using capillary tubes at 0, 30 and 60 min after insulin administration.

### Measurement of serum glucose, insulin and body weight

Serum glucose, insulin levels and body weight were measured at GD 0, 10 and 20. Non-fasting blood samples were obtained via tail venipuncture, and serum glucose level was determined by glucometer (Lifescan Surestep). Plasma insulin levels were quantified by UltraSensitive Mouse Insulin ELISA kit (ALPCO Diagnostics). Body weight was measured on a top-loading balance (Fisher Scientific).

### Liver dissection

Livers were dissected using previously established method [19]. On GD 10 after completing all other measurements, 6 out of 18 mice in each group were sacrificed to harvest the liver, while the remaining 12 females were allowed to complete pregnancy, and their offspring were also sacrificed after birth to harvest the liver. Mice were anesthetized with 100 mg/kg ketamine, 10 mg/kg acepromazine and 100 mg/kg xylazine. The abdominal cavity was opened and the portal vein exposed. A bolus of insulin (10 U/kg body weight in 100 μL saline) was injected into the portal vein. The liver was immediately excised and frozen at −80 °C until further analysis.

### Immunoblot and antibodies

Protein samples were extracted from liver homogenates in the presence of protease and phosphatase inhibitors. Protein concentration was quantified by BCA assay system (Bio-Rad, USA). Proteins were analyzed by 12 % SDS-PAGE and transferred to nitrocellulose membrane using semi-dry transfer unit (Bio-Rad). The membrane was then immersed in blocking buffer (PBS, 0.1 % Tween-20) with 5 % nonfat milk for 20 min and then incubated with appropriate primary antibody (1:1000 dilution in blocking buffer) overnight at 4 °C. After washes with blocking buffer, blots were incubated with appropriate HRP-conjugated secondary antibody (1:5000 dilution in blocking buffer; Life Technologies) for 20 min at room temperature, washed again with blocking buffer, and visualized using Luminata Forte Western HRP Substrate (EMD Millipore). AMPK (#2532), phosphor-AMPK (#2535), HDAC4 (#2072) and phosphor-HDAC4 (#3443) antibodies were purchased from Cell Signaling. Glucose-6-phosphatase (ab83690) and α-tubulin (ab125267) antibodies were purchased from Abcam.

### Liver glucose-6-phosphatase (G6Pase) activity

Microsomes were prepared from frozen liver samples as previously described [19]. G6Pase activity was measured on intact microsomes with 0.5, 2.5, and 10 mM of glucose-6-phosphate (G6P) as previously described [19]. Total protein was determined by the Bradford method. G6Pase activity was normalized by subtracting nonspecific phosphatase activity, which was determined using paranitrophenylphosphate.

### Statistical analysis

All values were presented as mean ± SEM, with number of independent experiments stated in respective figure legends. Statistical significance was analyzed using two tailed Student’s t-test between groups stated in respective figure legends. Differences were determined to be statistically significant when *P* < 0.05.

## Results

### RV relieved glucose and insulin intolerance in pregnant *db/+* mice

We first examined the effect of RV in the non-pregnant *db/+* mice, which usually do not present diabetic symptoms as previously reported [19]. By employing a glucose tolerance test in all three groups of non-pregnant mice, we confirmed that glucose and insulin levels were similar in wild type and pair-fed *db/+* mice, and RV didn’t cause any changes in either glucose or insulin levels (Fig. 1b and c).

Next we performed the same glucose tolerance test in all three groups of mice, during their pregnant state on GD 10. We found that, compared with wild type, pregnant *db/+* females exhibited prominent glucose intolerance, as indicated by significantly elevated glucose levels after the initial glucose injection (Fig. 1d, 30 and 60 min), and RV treatment was able to largely alleviate this glucose intolerance in the pair-fed *db/+* mice. In the same experiment, insulin levels of *db/+* mice was significant lower than that of wild type throughout the test (Fig. 1e, 0, 30 and 60 min), explaining the glucose intolerance phenotype. Importantly, RV administration restored insulin response to an indiscernible level as the wild type control mice.

Compared to glucose tolerance test, which assesses the combined outcome of glucose disposal and insulin secretion, the insulin tolerance test is a more direct measurement on the ability of insulin to rapidly stimulate glucose disposal [8]. Therefore we next performed an acute insulin tolerance test on GD 10 in all three groups of mice (Fig. 1f). As expected, the pair-fed *db/+* females exhibited significantly higher blood glucose levels than the wild type following the initial insulin injection, suggesting insulin resistance phenotype. Notably RV administration markedly reduced glucose levels in the pair-fed *db/+* mice, to similar levels as the wild type. Taken together, the above results indicated that RV was able to extenuate acute glucose and insulin intolerance in pregnant *db/+* mice.

**Fig. 2:**
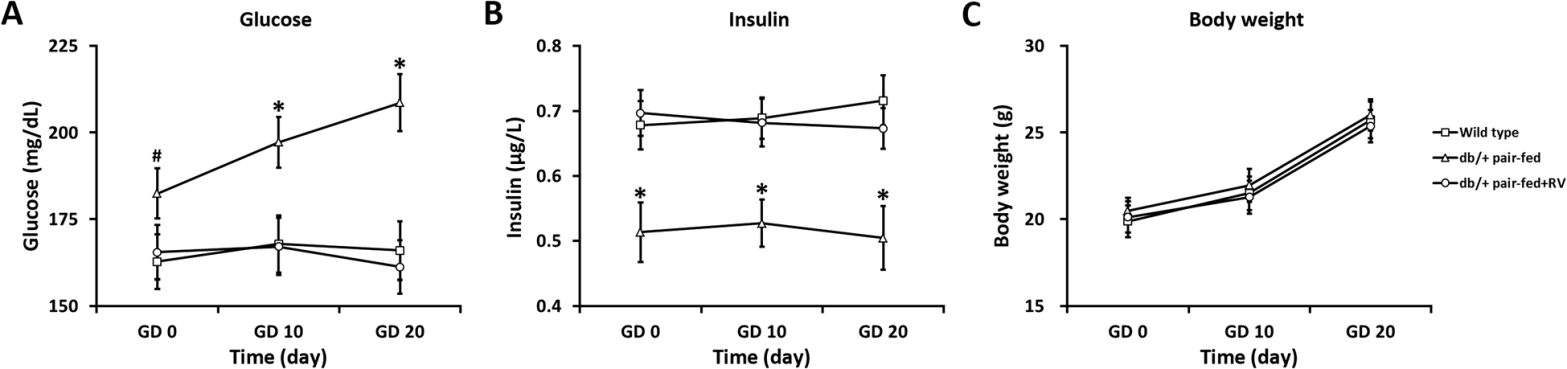
RV relieved GDM symptoms in pregnant *db/+* mice. Serum glucose (**a**) and insulin levels (**b**), and maternal body weight (**c**) were measured on gestation day (GD) 0, 10 and 20 in wild type, *db/+* pair-fed and *db/+* pair-fed + RV mice. Values represented mean ± SEM (*n* = 12). *P < 0.01, ^#^ *P* < 0.05, *db/+* pair-fed vs wild type and *db/+* pair-fed + RV mice at the same time point

We also measured the steady state levels of blood glucose and insulin levels throughout the pregnancy. We found that blood glucose levels of wild type females remained stable on GD 0, 10 and 20 (Fig. 2a). Whereas meanwhile, *db/+* females showed significantly elevated blood glucose levels, indicating the typical hyperglycemia symptom of GDM. However, blood glucose levels of *db/*+ females receiving RV stayed at the same as the wild type group through the entire duration of pregnancy, indicating the alleviating effect on hyperglycemia by RV. In a similar manner, steady state insulin levels in *db/+* mice were also significantly lower than wild type, which could be fully restored by RV administration (Fig. 2b). Maternal body weight gains in all three groups of mice were almost the same throughout the pregnancy (Fig. 2c).

### RV increased liver AMPK activation in pregnant *db/+* mice

**Fig. 3:**
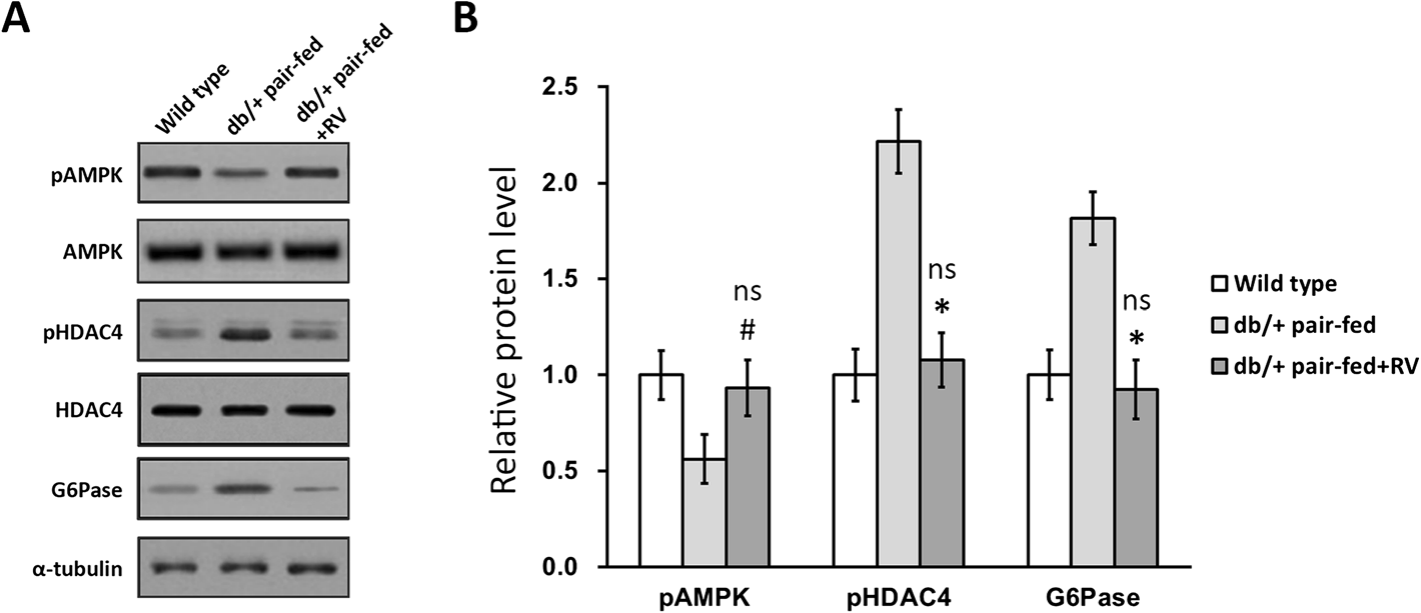
RV increased AMPK activation in pregnant *db/+* mice. Representative immunoblot (**a**) and quantification (**b**) of phosphorylated(p)-AMPK, total AMPK, pHDAC4, total HDAC4 and glucose-6-phosphatase (G6Pase) in pregnant mice liver on GD 10. Values represented mean ± SEM (*n* = 6). *P < 0.01, ^#^ *P* < 0.05, *db/+* pair-fed + RV vs *db/+* pair-fed mice. ns not significant, *db/+* pair-fed + RV vs wild type mice

As AMPK is likely to be a potential target of RV function in diabetes, we sacrificed 6 mice from each group at GD 10 and harvested their livers for immunoblot analysis. As shown in Fig. 3a, AMPK activation was found to be attenuated in db/+ pair-fed mice, contributing to higher HDAC4 activation, which in turn elevated G6Pase expression in the liver. Consistent with previous reports [15, 16], RV administration fully reversed the trend of activation profile of the related proteins, eventually reducing G6Pase levels to almost the same as in wild type (Fig. 3b).

### RV improved reproductive outcome of pregnant *db/+* mice

One of the adverse effects of GDM is decreased fetal survival, presented as fewer number of fetuses [20, 21]. Moreover in the same *db/+* GDM mouse model, body weight of offspring at birth was reported to increase by 5–8 % [7, 8]. Therefore we next investigated whether RV was able to improve fetal development of GDM female mice.

**Fig. 4:**
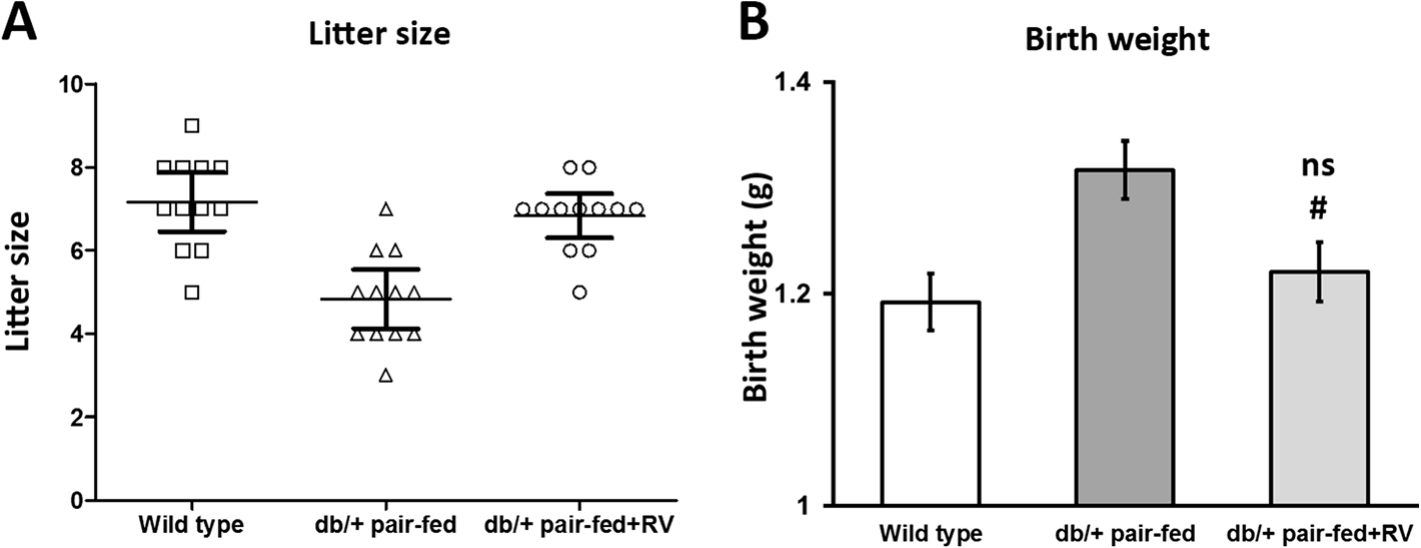
RV improved reproductive outcome of pregnant *db/+* mice. Litter size (**a**) and body weight at birth (**b**) of offspring born by each female mouse from wild type, *db/+* pair-fed and *db/+* pair-fed + RV experimental groups were recorded (*n* = 12). ^#^ *P* < 0.05, *db/+* pair-fed + RV vs *db/+* pair-fed mice. ns not significant, *db/+* pair-fed + RV vs wild type mice

Offspring number at birth was counted for each females from wild type, *db/+* pair-fed and *db/*+ pair-fed + RV groups (Fig. 4a). The 12 wild type female mice gave birth to 86 litters, whereas the 12 females from the *db/+* pair-fed group gave birth to only 58 litters. In contrast, 82 litters were born by the 12 *db/+* pair-fed + RV group of females. Furthermore we recorded the body weight of offspring at birth in the three groups (Fig. 4b), and found that, consistent with previous reports [7, 8], mean body weight of offspring born by *db/+* pair-fed mothers was significantly higher than those by wild type mothers, whereas body weight of offspring from *db/*+ pair-fed + RV group was almost the same as wild type control. Differences in reproduction outcome among these groups was not attributed to any specific GDM genetic condition of the fetus, since all of their offspring genotype composition followed Mendelian ratio (Additional file 1: Table S1). These results clearly indicated RV was able to improve the reproductive outcome of GDM female mice.

### RV increased liver G6Pase activity in offspring of *db/+* mice

**Fig. 5:**
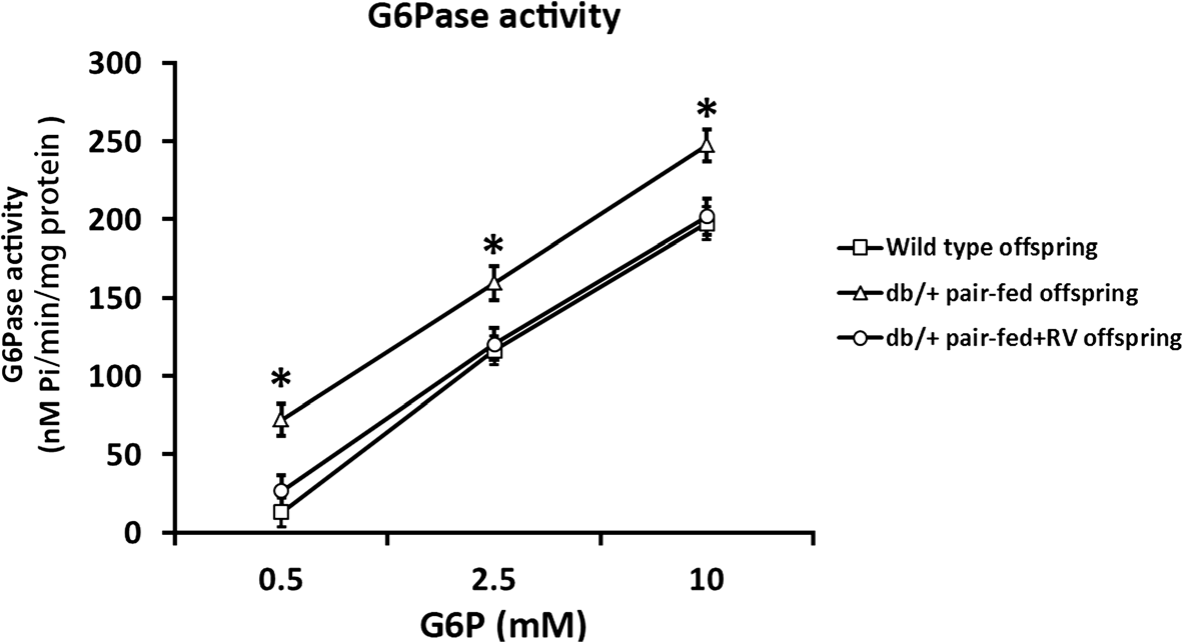
RV reduced glucose-6-phosphatase (G6Pase) activity in GDM offspring. G6Pase activity in liver of offspring born by wild type, *db/+* pair-fed and *db/+* pair-fed + RV mice were measured at birth (*n* = 12). *P < 0.01, *db/+* pair-fed vs wild type and *db/+* pair-fed + RV offspring

We speculate the reason for the increased body weight at birth of offspring from *db/+* mice could also be attributed to elevated liver G6Pase. Therefore we chose 12 offspring from each group of female mice, with the same genotype composition, and harvested their livers to assay for G6Pase activity (Fig. 5). As expected, G6Pase activity was significantly increased in the *db/+* pair-fed offspring, which could be reduced by RV administration in the *db/+* pair-fed + RV offspring. This result clearly indicated that RV also reduced the enzymatic capacity for glucose production in the fetus, likely through enhancing AMPK activation (Fig. 3).

## Discussion

GDM affects almost 10 % of pregnancies, and women suffering from GDM have higher risk of long-term type-2 diabetes [1]. Although increasing body of basic and clinical researches have been conducted on diabetes, very few of them were specifically tailored towards GDM, which took the approach of dietary intervention [22]. However, given the relative similar cause and symptoms between diabetes and GDM, substances such as RV in our current study with beneficial effect on diabetes are highly likely to exhibit similar functions in alleviating GDM. Indeed out results demonstrated that RV was able to significantly alleviate GDM symptoms such as hyperglycemia, insulin resistance, decreased fetal survival and increased body weight at birth in the *db/+* genetic mouse model.

Although previous study has found that RV could protect chicken embryos from high-glucose-induced oxidative stress and mal-development [23], our study is the first of its kind to report that RV exhibited alleviating functions in treating GDM in mammals. Furthermore, we investigated into the underlying molecular mechanism responsible for the observed effect of RV in GDM mouse model. We found that RV could increase the activation of AMPK in the liver of pregnant *db/+* mice, as well as their offspring. Our findings points to the function of RV to activate AMPK, which is consistent with previous report [24]. Moreover in mice deficient in AMPK catalytic subunits, RV failed to alleviate diabetic symptoms, further indicating the involvement of AMPK in RV-mediated effects [25]. In the liver, AMPK activation regulates glucose production, in that it inhibits HDACs, which in turn leads to down-regulated G6Pase expression and activity [18]. Our current work, together with those by Guo et al. and O’Tierney-Ginn et al. [15, 16], consistently pointed to the role of AMPK in the regulation of diabetes and GDM. In this context, AMPK could serve as a biomarker for the diagnosis and treatment of diabetic symptoms, which also warrants the search for other compounds that modulate AMPK activation.

Another major adverse symptom of GDM is fetal mal-development, which was reported in several studies using rodent models [5, 7, 8, 20, 21]. Reduced offspring number is one of the common phenotypes, as consistently observed in our study as well. On the other hand, diverse data on body weight of offspring at birth was observed in different models, higher in mice whereas lower in rats [26, 27]. These seemingly contradictory results were likely caused by either dietary or species variations. In our study the *db/+* genetic GDM females were fed with a diet higher in protein than standard diet [28], which might be the one of the reasons that offspring born by GDM mothers have nearly 10 % increase in body weight at birth, compared with those born by wild type mothers. Importantly, RV administration was able to fully rescue this defect in fetal development in *db/+* mice, likely through down-regulation of G6Pase activity in fetal livers. In this context, our results to the potential combination of a diet lower in protein content and RV to further improve reproductive outcome of GDM mice, at least in reducing the abnormally high body weight at birth of offspring.

In conclusion, our current study provided the first instance of using the natural compound RV as a therapeutic reagent to treat GDM in the *db/+* mouse model. RV administration significantly relieved hyperglycemia and insulin resistance in pregnant *db/+* female mice, as well as improved fetal development and reproductive outcome. Further analysis revealed that this effect of RV was mediated by elevated AMPK activation, which in turn inhibited HDAC4 phosphorylation, and eventually down-regulated G6Pase expression and activity. Findings in our study have further supported the potential therapeutic effect of RV in treating not only diabetes, but also alleviating GDM symptoms.

## Additional file

Additional file 1: Table S1. Genotype of offspring from three experimental groups of females. (DOCX 14 kb)

## Abbreviations

GDM: Gestational diabetes mellitus
RV: Resveratrol
AMPK: AMP-activated protein kinase
HDACs: Histone deacetylases
