# Supplementary material for: Resveratrol relieves gestational diabetes mellitus in mice through activating AMPK
Source: Reprod Biol Endocrinol. 2015 Nov 5;13:118. doi: 10.1186/s12958-015-0114-0 (PMC4635591; doi:10.1186/s12958-015-0114-0)
Supplement: Additional file 1: Table S1. — Genotype of offspring from three experimental groups of females. (DOCX 14 kb) [file 12958_2015_114_MOESM1_ESM.docx]

**Table S1.** Genotype of offspring from three experimental groups of females.

| Genotype | Wild type  offspring (n = 86) | *db/+* pair-fed  offspring (n = 58) | *db/+* pair-fed+RV offspring (n = 82) |
| --- | --- | --- | --- |
| +/+ | 86 (100%) | 16 (28%) | 23 (28%) |
| *db/+* | N.A. | 27 (47%) | 37 (45%) |
| *db/db* | N.A. | 15 (26%) | 22 (27%) |

N.A. not applicable. Percentages shown in brackets, and do not sum up to 100% due to rounding.
